# Mucosal Abnormalities in Children With Congenital Chloride Diarrhea—An Underestimated Phenotypic Feature?

**DOI:** 10.3389/fped.2020.00365

**Published:** 2020-07-29

**Authors:** Elena Kurteva, Keith J. Lindley, Susan M. Hill, Jutta Köglmeier

**Affiliations:** Great Ormond Street Hospital for Children NHS Foundation Trust, London, United Kingdom

**Keywords:** congenital chloride diarrhea, mucosa, inflammation, pathophysiology, IBD

## Abstract

**Objectives and Study:** Congenital chloride diarrhea (CCD) is a rare, autosomal recessive disorder caused by mutations in the *SLC26A3* gene encoding a transmembrane chloride/bicarbonate ion exchanger mainly expressed in the apical brush border of the ileal and colonic epithelium. Lifelong, secretory, chloride-rich diarrhea and hypochloremic, hypokalemic metabolic alkalosis are characteristic. Histological evidence of bowel inflammation is not typically described in CCD and has only been reported in a few patients.

**Methods:** We report four cases of CCD who received adequate resuscitation with appropriate replacement of their fecal salt and water losses. Three had associated inflammatory bowel changes at endoscopy. The index case of CCD who developed frankly bloodstained diarrhea aged 7 months was found to have histologically confirmed colitis at endoscopy. An electronic search of the hospital database to identify all patients with confirmed CCD was performed. A further three children underwent *de novo* diagnostic evaluation and treatment. A retrospective case note review was undertaken to determine the incidence and subtype of inflammatory bowel disease (IBD) by clinical, endoscopic, and histological means.

**Results:** Four children with genetically confirmed CCD were identified, two being female. The first girl had a granulomatous colitis with ulceration. She went into remission with a combination of steroids and azathioprine. Immunosuppression was subsequently discontinued without a further flare of colitis. A second girl was found to have patchy inflammatory changes in the small bowel and focal active colitis. A third patient, a boy, demonstrated mild inflammatory changes in the small bowel with apoptotic debris and mild inflammation in the colon. A fourth patient did not develop intestinal inflammation.

**Conclusion:** Our case series highlights the potential association of CCD with panenteric inflammation. While our cohort was small, CCD is rare and three out of four children referred to our tertiary referral center were affected. While early diagnosis and adequate salt replacement therapy are crucial in CCD management, the clinician should also be aware of bowel inflammation as a potential cause of failure of CCD therapy to control bowel symptomatology. Further insight is needed to understand the underlying patho-mechanism giving rise to bowel inflammation in this group.

## Introduction

Congenital chloride diarrhea (CCD) is a rare, autosomal recessive disorder, first described by Gamble et al. (1) in 1945. It is caused by mutations in the solute carrier family 26 member 3 (*SLC26A3* alias DRA) gene on chromosome 7q31 and is characterized by life-long secretory diarrhea (2). Disruption of the transport of chloride/bicarbonate (Cl^−^/HCO3^−^) across the ileal and colonic epithelium results in watery stools with an excess of chloride. Affected patients are at risk of life-threatening dehydration, and gastroenteritis may result in severe electrolyte disturbances. Most cases originate from Finland, Poland, and Arabic countries, where distinct founder mutations have been described (3). Patients from other parts of the world appear to have rarer homozygous or compound heterozygous mutations (4). Long-term outcome is encouraging, as long as fecal salt and water losses are adequately replaced (5).

Persistent voluminous watery diarrhea begins *in utero*, presenting with polyhydramnios and dilated intestinal loops (6). Other common features are preterm delivery, lack of meconium passage, abdominal distension, and failure to thrive. In the neonatal period, symptoms are marked with hyponatremia, hypochloremia, hyperbilirubinemia, later accompanied by hypokalemic metabolic alkalosis, and secondary hyperaldosteronism.

In infants, the diagnosis of CCD may be delayed, as the watery diarrhea may be mistaken for urine (7).

Early diagnosis and adequate therapy are essential to avoid the risk of severe hypovolemic dehydration and electrolyte abnormalities associated with a higher mortality and long-term complications, such as poor growth, mental impairment, hyperuricemia, and chronic kidney disease (5). Acute kidney injury in association with gastroenteritis and severe dehydration is common. Other associations include inguinal hernias, increased sweat chloride, spermatoceles, and reduced fertility in males. Intestinal inflammation has only been reported in a few cases (5, 8, 9). In a case study from Finland, the majority of children adjusted to the diarrhea over time and reported only a minor impact on daily life (5). In this cohort, a potential increased risk for inflammation of the gastrointestinal tract was noted, as three of their patients had an unspecified colitis or Crohn's disease (CD). The authors could however not demonstrate the underlying pathophysiology, and the incidence of inflammatory bowel disease (IBD) in this group of patients remains unknown.

We report four pediatric patients with genetically confirmed CCD identified at our institution. Three developed IBD-like manifestations clinically and had evidence of panenteric inflammation on histological investigation of bowel biopsies. We review the current literature and discuss possible causative factors of a link between CCD and IBD.

## Methods

Case 1 was the index case with confirmed CCD in infancy. Despite adequate salt substitution, she developed worsening diarrhea with blood in the 1st year of life and was diagnosed with colitis on endoscopy. She required steroids and azathioprine to induce and maintain remission. A subsequent electronic search of genetically confirmed patients with CCD diagnosed and managed at our institution was performed to understand if bowel inflammation was present in other children with CCD. A further three patients with CCD were identified, two of whom had bowel inflammation. The clinical and histological phenotype of their inflammatory bowel disorders is described, and relevant data of each case are summarized in Table 1.

**Table 1:** Summary of relevant data for each case presented.

	**Case 1**	**Case 2**	**Case 3**	**Case 4**
Birth history	Female infant born at 39/40 Antenatal polyhydramnios and echogenic bowel. Bowel obstruction suspected but ruled out after birth	Female infant born at 35/40 Consanguineous parents. Antenatal polyhydramnios. Low birth weight	Male infant born at 37/40 Consanguineous parents	Born at 36/40 to consanguineous parents. Antenatal polyhydramnios and suspected bowel obstruction
Presentation	Hyponatremia, hypokalemia, persistent metabolic acidosis, and elevated serum levels of renin/aldosterone in the first week of life. Initially thought to suffer from polyuria which was later found to be watery diarrhea. Commenced on electrolyte supplements and referred to Nephrology for suspected Bartter syndrome. Renal pathology was excluded, and elevated stool chloride (116 mmol/L) was detected. CCD was suspected and referral to pediatric Gastroenterology unit. CCD diagnosis was confirmed, and sodium chloride and potassium chloride supplements were adjusted with normalization of sodium balance and acid/base status. Required gastrostomy insertion as unable to take supplements by mouth	Delayed motor milestones at age 2 months Multiple hospital admissions from the age of 7 months with watery diarrhea and vomiting. Worsening renal function and development of chronic kidney failure associated with episodes of severe dehydration. Bartter's syndrome suspected at age 20 months due to persistent hypochloremic, hypokalemic metabolic acidosis. Poor response to indomethacin, loperamide, and sodium/potassium supplementation. High stool chloride detected	14% weight loss, hyponatremia, and hypokalemia in first week of life. Chronic diarrhea with recurrent dehydration and failure to thrive. Elevated fecal chloride	No evidence of bowel obstruction after birth. Normal suction rectal biopsy ruling out Hirschsprung's disease Parents first concerned about watery stool at age 2 years. Two admissions with dehydration Stool sample revealed elevated fecal chloride (144 mmol/L). CCD was suspected, and oral supplementation with sodium chloride and potassium chloride commenced
Genetics	Homozygous for c.2024_2026dup;p (lle675dup) mutation in the *SLC26A3* gene	c.1386G>A (W462X) mutation in the *SLC26A3* gene	c.1386G>A homozygous SLC26A3 mutation	c.559G>T (p.Gly187) nonsense mutation in the *SLC26A3* gene
Progress	Vomiting and worsening diarrhea from age 7 months. Initially, marginally elevated fecal calprotectin of 394 mg/kg (<50 mg/kg). Symptoms gradually progressed. Started to pass blood per rectum at age 10 months, and fecal calprotectin went up to 1,414 mg/kg	Persistent watery diarrhea and poor weight gain despite adequate supplementation of sodium and potassium chloride	Supplementation with sodium chloride and potassium chloride Referred at age 6 months in poor nutritional status requiring 8 weeks of parenteral nutrition due to poor enteral tolerance. Trial of butyrate supplementation failed to reduce high stool output	Remained well on supplementation with sodium and potassium chloride. No further hospital admissions and stabilization of stool output
IBD	Upper and lower gastrointestinal endoscopy showed chronic pancolitis with focal active inflammation and ulceration. After a primary immunodeficiency was ruled out, a course of prednisolone was commenced. Although symptoms improved, she had ongoing diarrhea with intermittent blood per rectum and persistently elevated fecal calprotectin. At age 15 months, second colonoscopy confirmed chronic inflammation throughout the colon, with granulomas and eosinophils	Upper and lower endoscopy revealed erythema and ulcers in colon. Histology revealed duodenitis and patchy focal active colitis	Upper and lower endoscopy aged 8 months: mild mucosal abnormalities with florid apoptotic debris in small bowel and mild inflammation of colon	Normal upper and lower endoscopy. No clinical, endoscopic, or histological evidence of IBD
Therapy	A second course of oral prednisolone was prescribed, and azathioprine was added to the medication. Bloody stools settled, and fecal calprotectin levels returned to normal	Supplementation of sodium chloride and potassium chloride together with calcium butyrate and amino acid-based feed Repeat OGD and colonoscopy 8 months later showed a mild chronic inactive gastritis only with resolved histological changes in small and large bowel	Weaned onto extensively hydrolyzed formula and intravenous fluids after 2 months of parenteral nutrition. Intravenous fluids stopped after 4 months. Repeat endoscopic assessment of the bowel was normal	Adjustment of sodium chloride and potassium chloride supplementation. Good weight gain. No IBD treatment required
Outcome	Azathioprine was discontinued at age 3 years following normal upper and lower endoscopy Currently, 4 years 6 months of age and well. On daytime supplementation with sodium chloride (15 mmol twice daily) and potassium chloride (7.5 mmol twice daily) and 400 ml of Dioralyte (two sachets) with added NaCl (53 mmol) and KCl (38 mmol) overnight (*via* gastrostomy)	Well at age 7 years on 20 mmol of sodium chloride (30% solution) three times a day and 30 mmol potassium chloride daily when family moved to another country. Now aged 10 and well	15 years of age. On normal diet and receiving 16 mmol of sodium chloride three times daily and 16 mmol of potassium chloride twice daily. Stable without clinical evidence of IBD	Transitioned well to the adult services at age 17 years. At the time of discharge on 20 mmol sodium chloride (30% solution) three times a day and 30 mmol potassium chloride three times daily. Currently 26 years of age and well

## Results

### Case 1

A female infant was born at 39 weeks' gestation. The pregnancy was complicated by polyhydramnios requiring a single drainage at 35 weeks. An antenatal ultrasound showed an echogenic bowel, raising the suspicion of an underlying intestinal atresia, which was subsequently ruled out after birth. She presented with hyponatremia, hypokalemia, persistent metabolic alkalosis, and elevated serum levels of renin/aldosterone in the first week of life. Initially, she was thought to suffer from polyuria which was later found to be watery diarrhea. She was commenced on electrolyte supplements and referred to Nephrology for suspected Bartter syndrome. Renal pathology was ruled out, and stool analysis revealed an elevated stool chloride of 116 mmol/L. CCD was suspected, and a referral to our pediatric Gastroenterology unit was made. Genetic testing confirmed a mutation in the *SLC26A3* (alias DRA) gene, and the child was homozygous for the c.2024_2026dup;p. (lle675dup) variant. She was managed with oral sodium and chloride supplements to maintain normal sodium balance and acid/base status. As the child struggled with oral medication, a gastrostomy was inserted.

**Figure 1:**
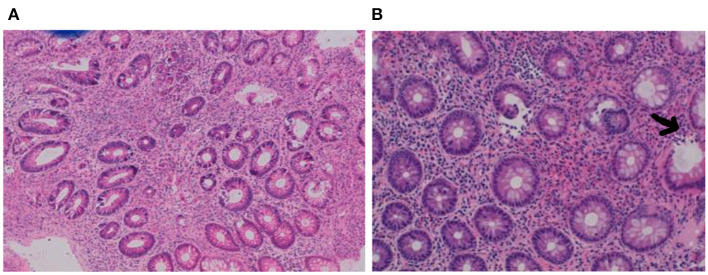
**(A,B)** Pretreatment biopsies demonstrating chronic pancolitis with focal active inflammation (arrow) and ulceration.

From the age of 7 months, she developed vomiting and worsening watery diarrhea. Her fecal calprotectin was initially considered to be marginally elevated at 394 mg/kg (adult normal values <50 mg/kg, but higher values are seen in the 1st year of life) in the context of a child with secretory diarrhea. Stool cultures and virology were negative. Her symptoms gradually progressed, and at the age of 10 months, she started to pass blood per rectum. The fecal calprotectin had increased to 1,414 mg/kg. A gastrointestinal panendoscopy was performed. The histopathology report showed chronic pancolitis with focal active inflammation and ulceration (Figures 1A,B). After a primary immunodeficiency was ruled out, a course of prednisolone was commenced.

Although her symptoms improved, she had ongoing diarrhea with intermittent blood.

Her fecal calprotectin was persistently elevated (1,380 mg/kg).

**Figure 2:**
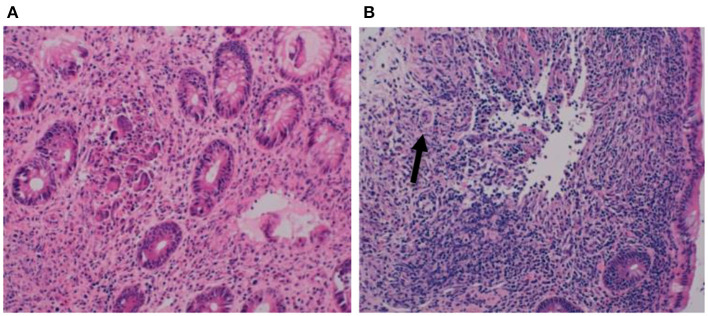
**(A,B)** Biopsies at second colonoscopy showing chronic pancolitis with granulomas (arrow) and eosinophils.

At the age of 15 months, a second colonoscopy was performed which confirmed chronic inflammation throughout the colon, with granulomas and eosinophils (Figures 2A,B).

She received a course of oral steroids with a good clinical response but a relapse of rectal bleeding after discontinuation of corticosteroids and persistently high fecal calprotectin (1,040 mg/kg). A further course of prednisolone was started, and azathioprine was added. The bloody stools settled, and the fecal calprotectin levels returned to normal (88 mg/kg). Azathioprine was discontinued at the age of 3 years following a normal gastrointestinal panendoscopy without a subsequent relapse of symptoms. She is currently 4 years 6 months of age and remains well on daytime supplementation with sodium chloride (15 mmol twice daily) and potassium chloride (7.5 mmol twice daily) and is receiving 400 ml of Dioralyte (two sachets) with added NaCl (53 mmol) and KCl (38 mmol) via gastrostomy overnight. She is thriving along the 50th centile both for height and weight on a normal diet.

### Case 2

A female patient was born at 35 weeks' gestation to consanguineous parents after her pregnancy was complicated with polyhydramnios from 6 months' gestation. She was noted to have low weight gain and delayed motor milestones at 2 months. From the age of 7 months, she required multiple hospital admissions with watery diarrhea and vomiting. Recurrent episodes of severe dehydration were subsequently associated with progressive worsening of her renal function and development of chronic kidney failure. At the age of 20 months, a diagnosis of Bartter's syndrome was suspected as she had a persistent hypochloremic, hypokalemic metabolic alkalosis. In addition, she developed athetoid movements, and an MRI of her brain revealed cerebral atrophy.

She was treated with indomethacin and loperamide for her diarrhea and also required sodium and potassium supplementation.

**Figure 3:**
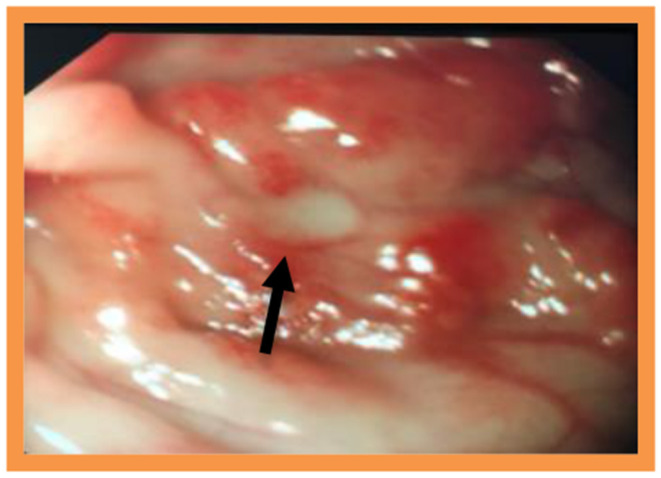
Macroscopic appearance of colon with erythema and occasional ulceration.

On this treatment, she gained weight adequately and was discharged home. She continued to have episodes of diarrhea with intermittent blood and/or mucus up to 12 times daily, prompting further investigations. Urine and stool samples revealed a low urinary chloride with a relatively high stool chloride of 114 mmol/L, which was incompatible with Bartter's syndrome and indicative of a diagnosis of CCD. Watery diarrhea and poor weight gain continued, and she was referred to our unit at age 3 years for an endoscopic examination of the upper and lower gastrointestinal tract. Macroscopically, there was erythema with occasional ulcers seen in the colon (Figure 3).

Histological examination revealed a patchy duodenitis with areas of villous blunting and increase in intraepithelial lymphocytes. The biopsies of the colon demonstrated a patchy focal active colitis with neutrophils in the lamina propria and cryptitis. A genetic study was performed and revealed a homozygous form of the c.1386G>A (W462X) genotype mutation in the *SLC26A3* gene, confirming the diagnosis of CCD. Adequate supplementation of sodium chloride and potassium chloride together with calcium butyrate was started, and the diet was supplemented with an amino acid-based feed. The child had a good clinical response with resolution of blood and mucus in the stools and good weight gain. Eight months later, a repeat endoscopic assessment showed mild chronic inactive gastritis with resolved histological changes in the small and large bowel. She remained well on oral supplementation with sodium and potassium chloride and continued to be followed up regularly until the age of 7 years when her family moved to another country. At the time of discharge, she received 20 mmol of sodium chloride (30% solution) three times daily and 30 mmol of potassium chloride daily. She is now 10 years old and remains stable.

### Case 3

A male infant born at 37 weeks to consanguineous parents presented with 14% weight loss, hyponatremia, and hypokalemia in the 1st week of life.

In the following months, he developed diarrhea associated with recurrent episodes of dehydration and failure to thrive. Serum electrolytes showed profound hypokalemia and hyponatremia. A stool sample sent for electrolytes demonstrated an elevated fecal chloride of 143 mmol/L. CCD was suspected. The genetic analysis confirmed the diagnosis and revealed a c.1386G>A homozygote mutation in the *SLC26A3* gene. The child was started on oral supplementation with sodium chloride and potassium chloride. He was referred to our unit at the age of 6 months for further investigations in poor nutritional status. He required 2 months of parenteral nutrition (PN) in view of poor feed tolerance. A therapeutic trial of butyrate supplementation failed to reduce his stool output. Upper and lower gastrointestinal endoscopies were performed. Histology of the biopsies revealed mild mucosal abnormalities with florid apoptotic debris in the small bowel and mild inflammation of the colon with an increase in lamina propria inflammatory cells including plasma cells and eosinophils. Repeat endoscopies after gut rest revealed mild inflammation in the colon only. He was eventually weaned onto an extensively hydrolyzed formula and intravenous fluids, as he continued to become dehydrated with feeds. He gradually improved, and intravenous fluids were stopped after 4 months. The repeat endoscopic assessment of the upper and lower GI tract was normal. He is now 15 years of age on a normal diet and is receiving 16 mmol of sodium chloride three times daily and 16 mmol of potassium chloride twice daily. He continues to open his bowels five to eight times a day with loose stools but no clinical evidence of IBD.

### Case 4

An 8-year-old boy was transferred to our institution at the age of 5 years with a history of chronic diarrhea and failure to thrive since infancy. He was born slightly preterm at 36 weeks' gestation to consanguineous parents. Antenatally, polyhydramnios was noted on ultrasound and bowel obstruction was suspected, which was not evident on a contrast imaging performed in the first week of life. A suction rectal biopsy confirmed the presence of ganglion cells, and Hirschsprung's disease was hence excluded.

His parents first noticed watery stools when he was 2 years old, and he had two admissions to hospital with dehydration.

Further investigations included a stool sample sent for electrolytes, which revealed an increased fecal chloride of 144 mmol/L and a lower stool sodium (<100 mmol/L). CCD was suspected, and oral supplementation with sodium chloride and potassium chloride commenced.

The diagnosis was confirmed genetically with DNA sequence analysis showing a homozygous pathogenic mutation {nonsense mutation, c.559G>T [p.(Gly187)]} detected in the *SLC26A3* gene. Gastrointestinal mucosal biopsies were unremarkable. He remained well on supplementation with improvement of weight gain and was transitioned to the adult services at age 17. At the time of discharge, he received 20 mmol of sodium chloride (30% solution) three times daily and 30 mmol of potassium chloride three times a day. He is now 26 years old and never developed clinical signs suggestive of inflammation of the bowel.

## Discussion

CCD is a rare autosomal recessive disorder due to mutations in the *SLC26A3* gene causing disruption of the Cl^−^/HCO3^−^ transport in the ileal and colonic epithelium. Only around 250 cases are published in the medical literature (2). Although previously described in a small number of affected patients, little is known about the incidence and natural history of intestinal inflammation in affected individuals (5, 8, 9). The underlying pathophysiology is poorly understood.

Sodium chloride (NaCl) is absorbed in the colon by the apical Na/H exchanger-3 (NHE3) and Cl^−^/HCO3^−^ exchanger SLC26A3 (DRA). In order for this process to work, NHE3 and DRA have to operate in close proximity to each other within an intact luminal and cytosolic microenvironment (10).

Disruption of this exchange mechanism will lead to inefficient salvage of water and solutes (Na^+^, Cl^−^, and short-chain fatty acids).

As a consequence, the fecal consistency will become very loose and the microbial ecosystem of the intestine can become disturbed (10).

The chronic intestinal inflammation seen in IBD is thought to be multifactorial and complex (11). A genetic predisposition, environmental factors, immune dysregulation within the intestinal mucosa, and dysbiosis are all held responsible.

The Na^+^/H^+^ exchangers (NHEs) are encoded by members of the SLC9 gene family and responsible for the electroneutral exchange of extracellular Na^+^ for intracellular H^+^ (12). NHEs are essential for the absorption of sodium and water and maintenance of intracellular pH. The pro-inflammatory cytokines interferon γ (INF-γ) and tumor necrosis factor-α (TNF-α), both involved in the pathophysiology of IBD, have been associated with a decrease in function and expression of NHE2 in human cacO-2/bbe cells (11, 13). In a rat model of trinitrobenzenesulfonic acid (TNBS)-induced colitis, NHE2 mRNA and protein expression in the colon was also significantly reduced, suggesting a link between NHE dysfunction and inflammation (14).

SLC26A3 protein is the most important anion transporter in the large bowel. SLC26A3-deficient mice present with similar life-threatening diarrhea seen in untreated CCD (15).

Interestingly, these mice also demonstrate hyperplasia of the surface epithelium in the colon and proliferation of crypts. Diarrhea-induced cell stress at the epithelial level may also contribute to the development of inflammation. In inflamed colonic mucosa, SLC26A3 is downregulated (7). The primary genetic defect in CCD may hence make affected individuals more vulnerable to the development of inflammation.

Increased intestinal permeability due to a damage of the protective layer of intestinal barrier is seen both in patients with CD and ulcerative colitis (UC). As a consequence, translocated luminal antigens activate the immune cascade within the mucosa. This in turn leads to a vicious cycle of worsening inflammation followed by an increase in diarrhea (16). The chronic diarrhea in CCD may have similar effects.

In addition to CCD, other transport defects are known to be associated with bowel inflammation. For example, cystic fibrosis (CF) is a multisystem disorder caused by mutations of the CF transmembrane conductance regulator (CFTR) protein that mostly affects the lungs, but also the pancreas, liver, kidneys, and intestine.

The CFTR gene is expressed in abundance throughout the intestine (17). Mouse models of CF have demonstrated that CFTR dysfunction leads to mucus accumulation within the intestinal lumen, disturbed motility, small bowel bacterial overgrowth, and inflammation with an abnormal innate immune response (18).

The domain interaction between SLC26A3 and the CFTR raises the possibility of CFTR modulation in CCD (2). CFTR may hence also play a role in the intestinal inflammationb seen in CCD.

Little attention has been given to the effect of acid–base status on the development of inflammation (19). The chloride-rich stool in CCD has a low pH. A complex association between changes in acid–base status and inflammation has been seen in critically ill patients, where a strong ion gap was found in those with higher plasma concentrations of interleukin 6 (IL-6), IL-8, IL-10, and TNF (20). A similar mechanism may contribute to the inflammation seen in the colon of patients with CCD.

## Conclusion

Our case series highlights a potential association of CCD with panenteric inflammation. Three of four children in this small series were affected. Early diagnosis and aggressive salt replacement therapy are crucial in CCD management. The clinician should, however, be aware of bowel inflammation as a potential cause of failure of conventional CCD therapy to control bowel symptomatology and the need for immunosuppression. The etiology accountable for bowel inflammation in this group of patients is likely multifactorial, and further insight is needed to understand the underlying patho-mechanism to allow for a timely diagnosis and targeted therapy in this already challenging group of patients.

## Data Availability Statement

The datasets analyzed in this article are not publicly available as they contain patient information. All relevant data important for the understanding and interpretation of the data are included in this article. Requests to access the datasets can be directed to Jutta.Koeglmeier@gosh.nhs.uk.

## Ethics Statement

Ethical review and approval was not required for the study on human participants in accordance with the local legislation and institutional requirements. Written informed consent to participate in this study was provided by the participants' legal guardian/next of kin. Written informed consent was obtained from the minor(s)' legal guardian/next of kin for the publication of any potentially identifiable images or data included in this article.

## Author Contributions

EK has made substantial contributions to the data collection from medical records and interpretation of data for the work and drafting the first version of the manuscript. KL has made substantial contributions to the conception and design of the work, interpretation of data, and proof reading of the final version of the manuscript. SH has made contributions to the data collection and interpretation of data and proof reading of the final version of the manuscript. JK holds the intellectual property of the work, was the scientific supervisor during the entire study period interpretation of data, substantial literature review, advised on the design of the study, and writing up the paper. She wrote and proof read the final version of the manuscript. All patients included in the study attended Great Ormond Street Hospital under the care of either JK, KL, or SH. Written consent to enter information into the study was taken in clinic by the parent. JK took part at drafting the work and final approval of the version to be published. All authors contributed to the article and approved the submitted version.

## Conflict of Interest

The authors declare that the research was conducted in the absence of any commercial or financial relationships that could be construed as a potential conflict of interest.
